# Comparative Pharmacokinetic Study of Taxifolin after Oral Administration of Fructus Polygoni Orientalis Extract in Normal and Fibrotic Rats by UPLC-MS/MS

**DOI:** 10.1155/2019/9348075

**Published:** 2019-12-31

**Authors:** Feili Wei, Li Guo, Yongsong Xu, Dexi Chen, Muxin Gong

**Affiliations:** ^1^School of Traditional Chinese Medicine, Capital Medical University, Beijing 100069, China; ^2^Beijing Key Lab of TCM Collateral Disease Theory Research, Beijing 100069, China; ^3^Beijing Institute of Hepatology, Beijing Youan Hospital, Capital Medical University, Beijing 100069, China

## Abstract

Fructus polygoni orientalis (FPO) is widely used in clinical practice in China, especially in treatment of liver diseases including viral hepatitis, liver fibrosis, and liver cirrhosis. However, its pharmacokinetic (PK) alterations in liver fibrotic rats have rarely been reported. To study whether taxifolin, one of the main flavonoids in FPO can be absorbed into blood after oral administration of FPO extract and to compare the differences in pharmacokinetic parameters of taxifolin to normal and liver fibrotic rats induced by porcine serum (PS), a UPLC-MS/MS method was developed and validated for determination of taxifolin in rat plasma using puerarin as the internal standard (IS). All validation parameters met the acceptance criteria according to regulatory guidelines. The results indicated that after treatment of rats with PS alone for 12 weeks, the liver fibrotic model group was built successfully. The taxifolin can be absorbed into the blood after oral administration of the FPO extract. The *C*_max_ of taxifolin was 1940 ± 502.2 ng/mL and 2648 ± 208.5 ng/mL (*p* < 0.05), the AUC_0∼*t*_ of taxifolin was 4949.7 ± 764.89 h·ng/mL and 6679.9 ± 734.26 h·ng/mL (*p* < 0.05), the AUC_0∼∞_ of taxifolin was 5049.4 ± 760.7 and 7095.2 ± 962.3 h·ng/mL (*p* < 0.05), and the mean residence time (MRT) of taxifolin was 2.46 ± 0.412 h and 3.17 ± 0.039 h (*p* < 0.05) in the normal and fibrotic model groups, respectively. These results confirmed that the pharmacokinetic parameters of taxifolin are altered in liver fibrosis, manifested as *C*_max_, AUC_0∼*t*_, AUC_0∼∞_, and the mean residence time (MRT). It suggested that it is essential to consider the characteristics of pharmacokinetics after oral administration of FPO in liver disease patients.

## 1. Introduction

Liver fibrosis is a common pathological stage in chronic liver injury caused by various factors that leads to the accumulation of extracellular matrix (ECM) and formation of fibrous scars [1–3].

Disruption of the liver structure by fibrous scars can result in the loss of hepatocyte cells and deregulation of normal liver functions and can ultimately develop into cirrhosis and liver failure [4, 5]. Animal experiments and clinical studies have shown that liver fibrosis, even early cirrhosis, is reversible [6, 7]. Combined treatment of etiology and fibrosis can accelerate the decline of liver fibrosis and promote liver regeneration [6, 8]. At present, many potential antifibrosis targets have been discovered through animal experiments, but most are still in the clinical trial stage [9–11].

As a traditional Chinese medicinal herb, FPO is the dried and mature fruit of *Polygonum orientale* L. It has been used in China for the treatment of various liver diseases such as hepatitis, liver fibrosis, and cirrhosis [12]. Taxifolin, also known as dihydroquercetin, is one of the most abundant flavonoids in FPO and has been listed as an indicator of quality for FPO in the Chinese Pharmacopeia 2015 edition [13, 14]. Studies have found that taxifolin possesses hepatoprotective properties, which are attributed to its ability to reduce oxidative stress and to inhibit the release of inflammatory mediators [15–17]. However, whether taxifolin can be absorbed into the blood after oral administration of FPO extract remains unclear.

The liver is a complex organ with the ability to influence drug pharmacokinetics [18]. Liver diseases can impact the PK of all drugs including absorption, distribution, metabolism, and elimination, especially when liver fibrosis is serious [19, 20]. A previous study showed that compared with healthy controls, the risk of exposure to pantoprazole in cirrhotic patients increased 5–8 times owing to decreased liver clearance [21].

The objective of this study was to establish and validate a sensitive and selective UPLC-MS/MS method and then to apply the method to pharmacokinetic comparison between normal and liver fibrotic rats after oral administration of FPO extract. We hope the results can provide meaningful reference information for FPO usage and for development of a safe and effective medication.

## 2. Experimental

### 2.1. Chemicals and Reagents

**Figure 1:**
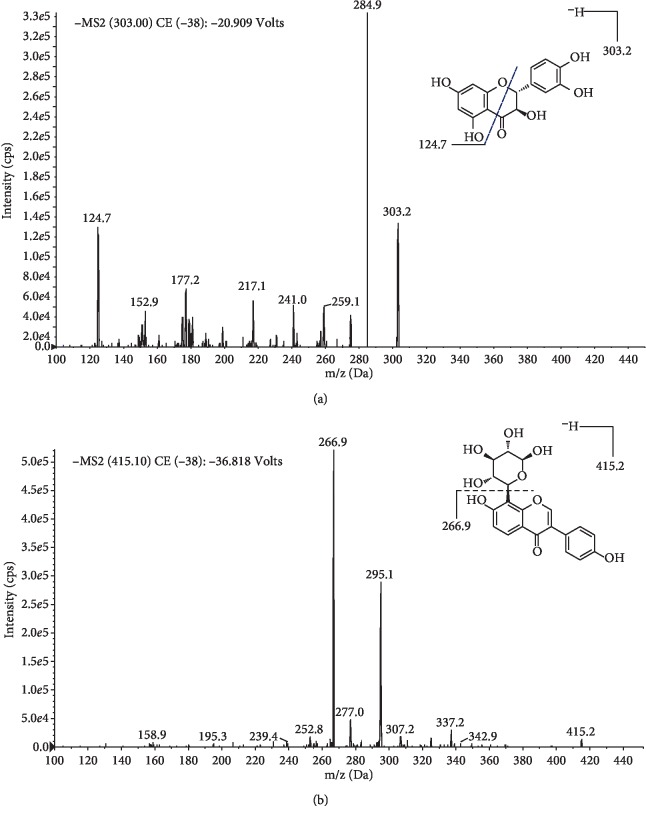
Full-scan product ion spectra of [M + H]^+^ ions and fragmentation schemes for (a) taxifolin and (b) puerarin (internal standard).

The FPO, batch no. 14053001, was purchased from the Beijing Tongrentang Pharmacy. After being crushed, FPO was refluxed three times by 70% (v/v) ethanol (2 h each at 70°C). After filtration, the alcohol extract was concentrated under reduced pressure at 45°C and then vacuumed to dryness at 80°C. Finally, the FPO extract was obtained as a brown powder. The porcine serum was obtained from Gibco (Gibco, New Zealand). The rat liver function and serum liver fibrosis indexes test kits were obtained from Nanjing Jiancheng Bioengineering Institute (Nanjing, China). The qRT-PCR reagent was obtained from TAKARA Bio Inc (DaLian, China), and the primer sequence was as described previously [22]. The reference standard of taxifolin (purity ≥98%, batch no. 111816-201102, chemical structure shown in Figure 1(a), stored at 4°C) and reference standard of puerarin (IS, purity ≥98%, batch no. 110752-201615, chemical structure shown in Figure 1(b), stored at 4°C) were both obtained from National Institutes for Food and Drug Control (Beijing, China). HPLC-grade acetonitrile and methanol were purchased from Fisher Scientific (Fair Lawn, NJ, USA). The other chemicals and reagents were of the highest grade commercially available.

### 2.2. Animals

Forty-two male Wistar rats (200 ± 20 g) were obtained from Beijing Vital River Laboratories Co., Ltd (Beijing, China). Following acclimatization for 1 week after arrival, thirty-six rats were used for evaluation of the efficacy and mechanism of FPO extract (data will show in another articles). Six rats were used in this comparative pharmacokinetic study. The six rats were randomly divided into normal and fibrotic groups (*n* = 3). The fibrotic group received 0.5 mL porcine serum twice a week for 12 weeks via intraperitoneal injection. At the same time, rats in the normal group were administered with the same volume of saline. The animals were housed in a temperature- and humidity-controlled environment with a 12 : 12 light-dark cycle and given free access to food and water. The ethics review was approved by the ethics committee of Capital Medical University with the ethics number AEEI-2014-128 (Beijing, China). In order to examine whether treatment of rats with PS alone for 12 weeks could induce hepatic fibrosis, liver function, and serum liver fibrosis indexes were detected by ELISA, the mRNA expression level of aSMA, Collagen1A1, and Collagen3A1 in liver tissues was measured by qRT-PCR, and liver condition was assessed by haematoxylin and eosin (H&E) and Masson's trichrome staining.

### 2.3. Chromatographic Conditions

A UPLC system equipped with an Agilent 1290 Bin Pump and a 1290 autosampler along with a 1290 TCC column oven (Agilent Technologies, Santa Clara, USA) were used in the study. Chromatographic separation was performed on an Agilent SB-C18 column (50 mm × 2.1 mm, 1.8 *μ*m) with a gradient elution by a mobile phase consisting of water containing 0.3% acetic acid (A) and acetonitrile containing 0.1% formic acid (B), which were filtered through a 0.45 *μ*m membrane filter and then degassed ultrasonically for 10 min. The gradient was as follows: 0.00 min 10% B, 0.50 min 10% B, 1.00 min 70% B, 3.00 min 98% B, 3.01 min 10% B, and 5.00 min 10% B, with a flow rate of 0.30 mL/min. The injection volume was set to 1 *μ*L. An API 6500 Qtrap mass spectrometer (Applied Biosystems/MDS Sciex, Concord, ON, Canada) was coupled with the UPLC system via a Turbo Ionspray ionization interface. The ESI source was operated in negative mode, and the curtain gases GS1 and GS2 were set at 20, 55, and 55 psi, respectively, following optimization of the setting parameters. The source temperature was set to 550°C, and the ionspray needle voltage was −4500 V. The mass spectrometer was operated at unit resolution for Q1 and low resolution for Q3 in the multiple reaction monitoring (MRM) mode, with a dwell time of 150 ms per multiple reaction monitoring channel (msec). The main MS parameters for taxifolin and puerarin (IS) are shown in Table 1. Analyst Data Acquisition and Processing software (Version 1.6.2, Applied Biosystems/MDS Sciex, Concord, ON, Canada) was used to collect and analyze the data.

### 2.4. Calibration Standards and Quality Control Samples

Stock solutions of taxifolin were prepared in ultrapure methanol at 2 mg/mL, and then a series of standard working solutions was prepared by diluting the appropriate amount of stock solution with ultrapure methanol to concentrations of 10–5000 ng/mL. A total of 1 mg/mL of IS solution was prepared in methanol : acetonitrile (50 : 50, v/v). The calibration standards were prepared by spiking 5 *μ*L of the corresponding taxifolin working solutions into 50 *μ*L of blank rat plasma to yield concentrations of 10, 50, 100, 500, 1000, 2000, 4000, and 5000 ng/mL. Quality control (QC) samples at low, medium, and high levels were prepared in the same way as the calibration samples to reach concentrations of 50, 1000, and 4000 ng/mL. All the calibration and QC samples were freshly prepared before analysis and stored at 4°C.

### 2.5. Sample Preparation

The QC samples, calibration standards samples, blank plasma samples (plasma samples got from the same batch of rat without any administration), and experimental rat samples were extracted using a protein precipitation procedure. First, these samples were thawed at room temperature for about 30 min and vortexed for 30 s. Aliquots of 50 *μ*L rat plasma were mixed with 5 *μ*L of methanol (or standards or QC solutions) and 200 *μ*L of IS solution (200 ng/mL puerarin in methanol : acetonitrile (50 :50, v/v). After being vortexed for 1 min and then centrifuged at 12,000 ×g for 10 min, aliquots of 130 *μ*L supernatants were transferred to UPLC vials. An aliquot of 1 *μ*L of sample was then injected into the UPLC-MS/MS.

### 2.6. Method Validation

The method was validated in compliance with the International Guidelines from the U.S. Department of Health and Human Services Food and Drug Administration (US FDA) [23–25].

#### 2.6.1. Selectivity

The specificity and selectivity of the UPLC-MS/MS method were evaluated by comparing six blank plasma samples from different sources with the corresponding spiked QC. The normal and fibrotic rat plasma samples were extracted after oral administration.

#### 2.6.2. Linearity

Calibration curves were acquired through analysis of eight standards in plasma samples and plotting of the peak-area ratio of the taxifolin and IS (puerarin) versus the corresponding taxifolin concentrations (10, 50, 100, 500, 1000, 2000, 4000, and 5000 ng/mL).

#### 2.6.3. Precision and Accuracy

To determine the intraday precision of the method, three plasma samples with concentrations of 10, 1000, and 4000 ng/mL were analyzed six times on the same day. To determine the interday precision and the accuracy, other three plasma samples with concentrations of 10, 1000, and 4000 ng/mL were run on each of three different days.

#### 2.6.4. Extraction Recovery and Matrix Effect

The matrix effects were expressed as the mean of the peak-area ratios of the blank plasma samples spiked with taxifolin after protein precipitation divided by the injected working solution with taxifolin at the same QC concentration.

#### 2.6.5. Stability

The stability of taxifolin in rat plasma was evaluated using three concentrations of QCs in triplicate. The stability of the prepared plasma samples was assessed after (A) incubating the samples at room temperature for 24 h followed by (B) three freeze-thaw cycles and (C) storage at −80°C for a month.

### 2.7. Application to Pharmacokinetics Comparison

The FPO extract was prepared by dissolution in distilled water. All rats were fasted for 12 h with free access to water before the experiments. The fibrotic rats were orally administrated with the FPO extract at a dose of 1.23 g/kg (equivalent to 52.5 mg/kg for taxifolin). The normal rats were administrated with the same volume of saline. Approximately, 300 *μ*L of blood samples were collected from the suborbital vein into a heparinized 1.5 mL centrifuge tube at 0.083, 0.167, 0.33, 0.50, 1.0, 2.0, 4.0, 8.0, 12, and 24 h after oral administration.

### 2.8. Statistical Analyses

All results were expressed as the arithmetic mean plus standard deviation (SD) and analyzed using SPSS 17.0 statistical software (SPSS Inc., Chicago, IL, USA).The analysis of variance (ANOVA) was used for comparison between groups. DAS Version 2.0 (Chinese Pharmacological Society, Beijing, China) was employed to analyze pharmacokinetic parameters including half-life (*t*_1/2_), maximum plasma time (*T*_max_) and concentration (*C*_max_), area under the concentration-time curve (AUC_0–*t*_ and AUC_0–∞_), clearance (CL), steady-state volume of distribution (*V*_Z_), and mean residence time (MRT) by noncompartmental methods. A value of *p* < 0.05 was considered statistically significant.

## 3. Results

### 3.1. Optimization of UPLC-MS/MS Conditions

**Figure 2:**
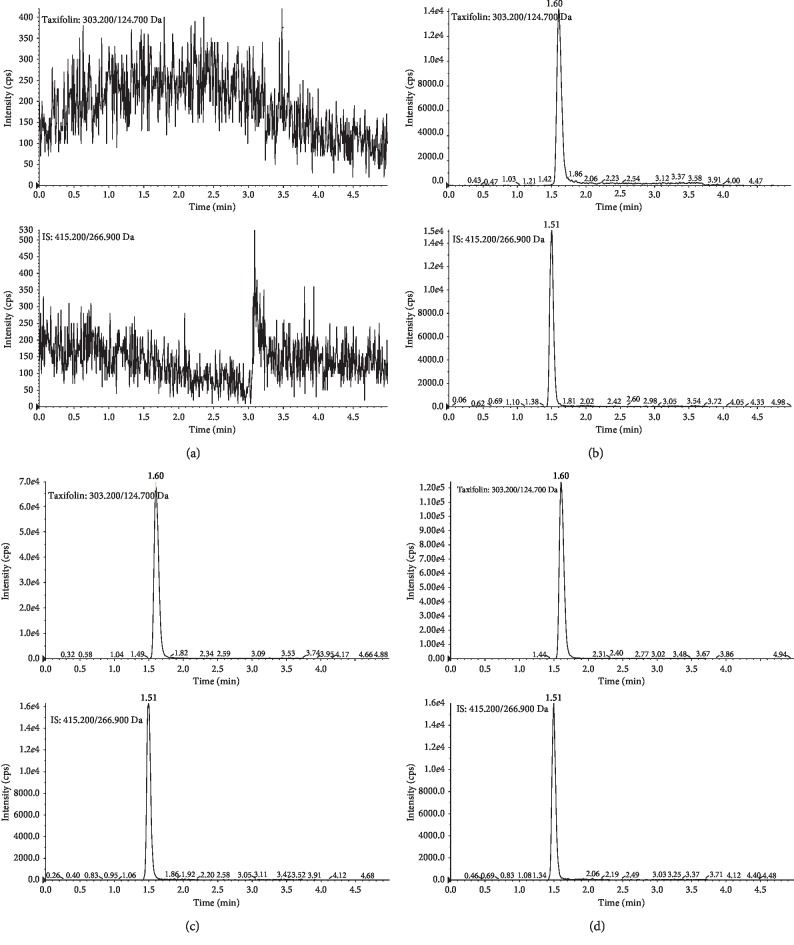
Typical chromatograms of (a) blank rat plasma; (b) blank rat plasma spiked with taxifolin (10 ng/mL, LLOQ) and IS; and (c) an normal rat plasma sample collected at 1 h after oral administration of 1.23 g/kg extract of FPO; (d) a model rat plasma sample collected at 10 min after oral administration of 1.23 g/kg extract of FPO.

This study first described the development of a sensitive and specific UPLC-MS/MS assay for the determination of taxifolin concentrations in rat plasma after oral administration of FPO extract. The full-scan product ion mass spectra of taxifolin and puerarin (internal standard; IS) are shown in Figure 1. Mass chromatograms of taxifolin and IS obtained by extraction of blank rat plasma, blank plasma spiked with taxifolin and IS, and actual unknown plasma samples obtained in rats after oral administration of FPO extract are shown in Figure 2. The chromatographic run time for the extracted plasma samples was 5.0 min. The retention times for taxifolin and IS were 1.60 and 1.51 min, respectively. The chromatograms showed baseline separation of taxifolin and the IS without any interference from endogenous plasma components.

### 3.2. Characteristics of Liver Fibrosis in Model Group

#### 3.2.1. Liver Function and Serum Liver Fibrosis Indexes after PS Treatment for 12 Weeks

Compared with the normal group, the liver function including ALT and AST showed a significantly elevated value (*p* < 0.05). The serum liver fibrosis indexes level of hyaluronic acid (HA), laminin (LN), type IV collagen (IV-C), and type III procollagen (PCIII) in the model group were significantly higher than in the normal group, and the results were shown in Table 2.

#### 3.2.2. The mRNA Expression Level of aSMA, Collagen1A1, and Collagen3A1 in Liver Tissues after PS Treatment for 12 Weeks

**Figure 3:**
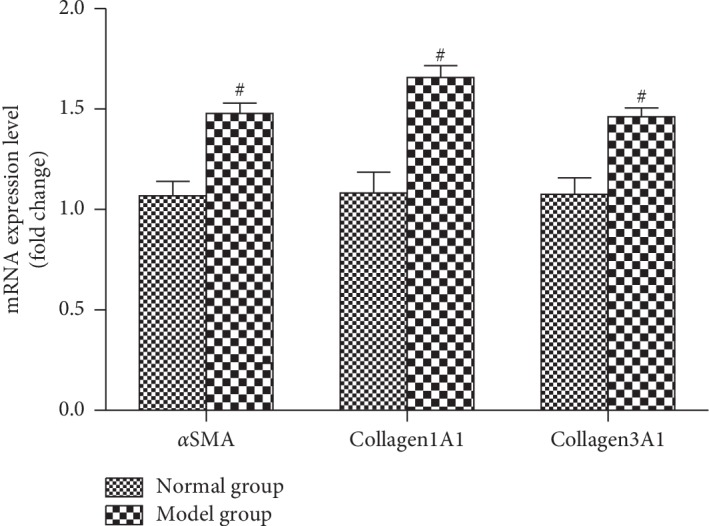
The fold change of mRNA expression level of 3 fibrosis-related genes including aSMA, Collagen1A1, and Collagen3A1 in liver tissues detected by qRT-PCR after PS treatment for 12 weeks. Data are expressed as mean ± SD(*n* = 3). ^#^*p* < 0.05.

As demonstrated in Figure 3, the fold change of mRNA expression level of 3 fibrosis-related genes include aSMA, Collagen1A1, and Collagen3A1 in liver tissues was significantly upregulated when compared with the normal live tissues (*p* < 0.05).

#### 3.2.3. Histopathological Manifestation in PS-Induced Liver Fibrosis

**Figure 4:**
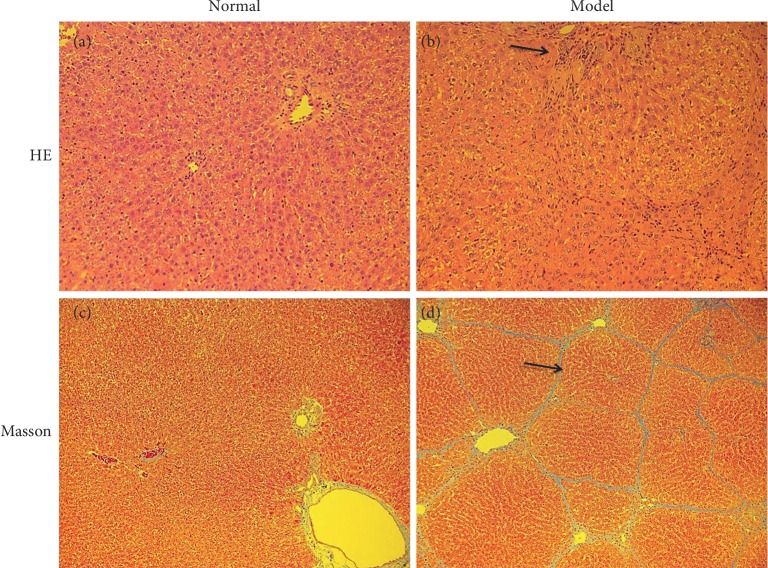
Photomicrographs of liver sections stained by (H&E) and Masson's trichrome staining (magnification 200x). Representative histological images of H&E stained rat liver tissue in the (a) normal group (normal), (b) PS-induced fibrotic group (model), respectively. Arrows indicate inflammatory cells infiltration. Representative histological images of Masson's trichrome stained rat liver tissue in the liver tissue in the (c) normal group (normal), (d) PS-induced fibrotic group (model), respectively. Arrows indicate the collagen fibers between the portal region and pseudolobules.

The haematoxylin and eosin (H&E) and Masson's trichrome staining results showed that in normal rat groups, no obvious hepatocellular injury was found, in contrast, inflammatory cells infiltration, necrosis were clear in the model group. The Masson's trichrome staining showed that thick fibrotic septa connecting portal tracts, delimiting the classic liver lobule, and the hepatic lobules were encysted and separated by collagen bundles. The degree of rat liver fibrosis determined by microscopy at 12 weeks was at pathologic grading III [26]. There were no obvious pathological changes observed in the normal groups. These results are shown in Figure 4.

### 3.3. Method Validation

#### 3.3.1. Specificity

The typical chromatograms of a blank plasma sample, an LLOQ sample, and *in vitro* plasma samples after administration of taxifolin are presented in Figure 2. Clearly, there was no significant endogenous interference at the retention times of taxifolin and IS, which indicates that the assay was selective.

#### 3.3.2. Linearity and LLOQ

The linear ranges of taxifolin in rat plasma ranged from 10 to 5000 ng/mL. The calibration curve for taxifolin had a correlation coefficient (*r*) of 0.995 or better. The lower limit of quantification (LLOQ) of taxifolin was 10 ng/mL.

#### 3.3.3. Precision and Accuracy

The intraday and interday precisions were defined as relative standard deviation (%RSD) with criteria of less than 15%; the accuracy was assessed by comparing the measured concentration with its nominal value using a criterion of ±15% for all QC samples. The results are summarized in Table 3.

#### 3.3.4. Extraction Recovery and Matrix Effect

The extraction recovery and matrix effect results are summarized in Table 4. A mean percentage matrix effect value of 95.9% for taxifolin was calculated and found to be independent of taxifolin plasma concentration and rat plasma lot. This result is in agreement with international guidelines and indicates low ion suppression.

#### 3.3.5. Stability

The described stability data are summarized in Table 5. The results indicated that taxifolin at the three concentrations tested had acceptable stability after storage at room temperature for 24 h, three cycles of freeze-thaw, and −80°C for 1 month with % RSD values of less than 15%.

### 3.4. Pharmacokinetic Parameter Comparison

**Figure 5:**
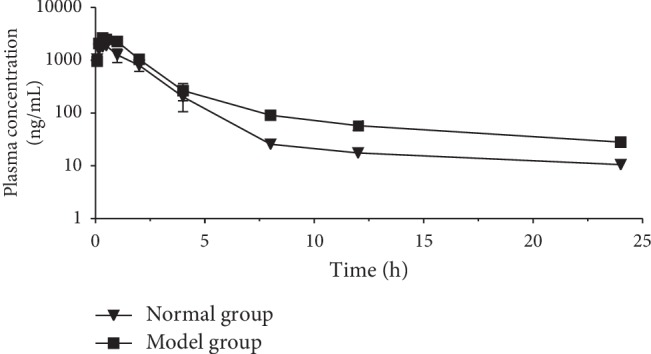
Mean plasma concentration-time profiles of taxifolin determined by the UPLC-MS/MS method after oral administration of FPO extract to rats. Each point represents the mean ± SD (*n* = 3).

This sensitivity and specificity method was applied to the pharmacokinetic study. The plasma concentration-time profiles of taxifolin in rats are shown in Figure 5. The main pharmacokinetic parameters for normal and fibrotic rats are summarized in Table 6. The peak plasma concentration (*C*_max_), area under the plasma concentration-time curve from time zero to *C*_max_ (AUC_0–*t*_)_,_ area under the plasma concentration-time curve from time zero to infinity (AUC_0–∞_), and mean residence time (MRT) in fibrotic rats were markedly increased. The *C*_max_ was 1940 ± 502.2 ng/mL and 2648 ± 208.5 ng/mL, respectively; the AUC_0∼*t*_ was 4949.7 ± 764.89 h ng/mL and 6679.9 ± 734.26 h ng/mL (*p* < 0.05); the AUC_0∼∞_ was 5049.4 ± 760.7 and 7095.2 ± 962.3 h ng/mL (*p* < 0.05); and the MRT of taxifolin was 2.46 ± 0.412 h and 3.17 ± 0.039 h (*p* < 0.05).

## 4. Discussion

Polygonum plants contain a variety of bioactive substances, mainly flavonoids, which have biological activities including scavenging of free radicals, antioxidant activity, and antitumor activity [27]. Taxifolin and quercetin are two important flavonoids in FPO [28, 29]. Studies have shown that the concentration of taxifolin is about ten times higher than that of quercetin in fructus polygoni orientalis [29, 30]. In our preliminary experiment, after oral administration of FPO extract, the taxifolin in plasma was much higher than that of quercetin (data not shown). Therefore, in this study, we mainly detected and compared the absorption of taxifolin into the blood and the pharmacokinetic changes in normal and liver fibrosis rats after oral administration of FPO extract. A UPLC-MS/MS method for quantification of taxifolin in rat plasma was developed and validated. The method showed adequate quantitative ranges, selectivity, linearity, accuracy, and precision. The recoveries and the matrix effects were suitable for quantitation. In brief, this method is suitable for the PK measurement.

The results showed that taxifolin can be absorbed into the blood after oral administration of FPO extract in both normal and hepatic fibrotic model rats. A previous study showed that after the rabbits were given different doses of taxifolin, it could be absorbed into blood, and *T*_max_ was consistent with our results. However, there were some differences in other pharmacokinetics, including *t*_1/2_, *C*_max_, AUC_(0⟶*t*)_, and AUC_(0⟶∞)_, which were all higher in our results [31]. The causes for these differences may be as follows: first, the animal models are inconsistent between the two studies, which may influence the absorption process of taxifolin. Second, the ingredients were not the same, and it is not clear whether there are some interactions between the compounds in FPO. Therefore, in clinical practices, the FPO is usually used in treatment of liver diseases instead of taxifolin alone. Taxifolin is one of the most effective flavonoids in silymarin and is a powerful hepatoprotective agent [32]. We inferred that taxifolin should be the most effective component in the FPO extract. The presence of liver injury has a significant impact on pharmacodynamics and pharmacokinetics [33, 34]. In this study, porcine serum-induced hepatic fibrotic model rats were built successfully after 12 weeks treatment. The PS-induced rat model is characterized by minor hepatocyte damage but intense immune response, and the mechanisms of fibrogenesis are similar to those of hepatic diseases in humans, especially viral hepatitis, which is one of the main causes of liver fibrosis in China [22, 35–38]. Our results indicate that liver fibrosis significantly altered the PK of taxifolin *in vivo* after oral administration of the FPO extract. The pharmacokinetic parameters of single-dose carvedilol changed in hepatic fibrosis when a CCl4 hepatic fibrosis model was used to study the antifibrosis and pharmacokinetic effects of carvedilol, which are manifested as delayed clearance and drug accumulation. This is thought to result from the decrease of CYP2D6 expression in the hepatic blood flow and liver [39, 40]. The *C*_max_ and AUC alteration in this study might have been affected by changes in drug absorption, and the delayed MRT might have been the result of drug clearance [41, 42].

## 5. Conclusion

In conclusion, we found that taxifolin can be absorbed into the blood and that hepatic fibrosis affects the pharmacokinetics of taxifolin after oral administration of the FPO extract. Therefore, personalized dosage adjustment should be considered in clinical practice, especially in patients with serious liver disease conditions.

## Figures and Tables

**Table 1:** MS parameters^a^ of taxifolin and puerarin (IS) determined by UPLC-MS/MS.

MS parameters	Taxifolin	Puerarin (IS)
Parent ion (*m*/*z*)	303.2	415.2
Product (*m*/*z*)	124.7	266.9
DP (V)	−120	-130
CE (eV)	−20.909	-36.818
CXP (V)	−13	
Ionspray (V)	−4500	
CUR (psi)	20	
GS1 (psi)	55	
GS2 (psi)	55	
CAD (psi)	Medium	
Source temperature	550	

^a^DP: declustering potential; CE: collision energy; CXP: cell exit potential; CAD: collision-activated dissociation.

**Table 2:** Liver function and serum liver fibrosis indexes after PS treatment for 12 weeks (*n* = 3).

Characterize	ALT (IU/L)	AST (IU/L)	HA (pg/mL)	LN (ng/mL)	IV-C (ng/mL)	PCIII (ng/mL)
Normal	37.10 ± 9.45	97.96 ± 11.33	247.09 ± 15.72	5.74 ± 1.58	34.76 ± 1.11	11.71 ± 0.84
Model	72.95 ± 21.10	173 ± 59.29	291.18 ± 40.44	13.56 ± 1.85	41.53 ± 1.53	17.45 ± 3.47
*p* value	<0.05	<0.05	<0.01	<0.01	<0.01	<0.01

**Table 3:** Intra- and interday precision and accuracy of taxifolin in rat plasma (intraday: 6 replicates at each concentration; interday: 18 replicates at each concentration).

Concentration (ng/mL)	Intraday (*n* = 6)			Interday (*n* = 6 × 3)		
	Measured concentration (ng/mL)	Precision (RSD, %)	Accuracy (RE, %)	Measured concentration (ng/mL)	Precision (RSD, %)	Accuracy (RE, %)
10	9.3 ± 1.1	11.8	7.5	8.8 ± 1.1	12.5	12.0
50	47.9 ± 3.3	6.9	4.1	48.6 ± 3.5	7.1	2.8
1000	981.8 ± 42.9	4.4	1.8	999.2 ± 42.9	4.3	0.1
4000	3886.4 ± 106.2	2.7	2.8	3821.4 ± 117.4	3.1	4.5

**Table 4:** The mean recoveries and matrix of taxifolin and the internal standard in rat plasma (*n* = 6).

Spiked concentration (ng/mL)	Recovery (%)	RSD (%)	Matrix effect (%)	RSD (%)
50	93.5	5.1	97.8	6.6
1000	94.6	4.5	95.9	5.6
4000	96.2	2.4	97.9	3

**Table 5:** Stability of taxifolin in rat plasma under a variety of storage conditions (*n* = 3).

Spiked concentration (ng/mL)	Room temperature for 24 h		Three freeze-thaw cycles		−20°C for 1 month	
	Measured concentration (ng/mL)	RE (%)	Measured concentration (ng/mL)	RE (%)	Measured concentration (ng/mL)	RE (%)
50	49.7 ± 4.9	−0.7	50.9 ± 1.1	1.7	51.1 ± 2.2	2.1
1000	1021.7 ± 41.8	2.2	963.4 ± 45.7	−3.7	949.3 ± 34.4	−5.1
4000	3846.3 ± 99.5	−3.8	3878.3 ± 89.9	−3.0	3766.7 ± 120.2	−5.8

**Table 6:** Main pharmacokinetic parameters of taxifolin in rats determined after oral administration of 1.23 g/kg FPO extract (*n* = 3, mean ± SD).

Parameters	Unit	Normal group	Model group	*p* value
*t*_1/2_	h	14.66 ± 5.11	9.77 ± 4.19	0.269
*T*_max_	h	0.44 ± 0.096	0.33 ± 0	0.116
*C*_max_	ng/mL	1940 ± 502.2	2648 ± 208.5	0.044^#^
AUC_(0⟶*t*)_	h·ng/mL	4949.7 ± 764.89	6679.9 ± 734.26	0.048^#^
AUC_(0⟶∞)_	h·ng/mL	5049.4 ± 760.7	7095.2 ± 962.3	0.045^#^
F	%	5.778 ± 4.01	5.64 ± 2.77	0.963
Vz	mL/kg	73912 ± 45956.3	48327 ± 15259.3	0.428
Cl	mL/h	3382.4 ± 740.8	3538 ± 533.7	0.856
Ke	1/h	0.0524 ± 0.0219	0.0799 ± 0.0321	0.287
AUCINF_pred	h·ng/mL	4513 ± 1580.7	7073.7 ± 947.6	0.074
MRTlast	h	2.46 ± 0.412	3.17 ± 0.039	0.04^#^

^#^ *p* < 0.05.

## Data Availability

The data used to support the findings of this study are included within the article.
